# The effect of training program to reduce needlestick injuries

**DOI:** 10.1186/1753-6561-5-S6-P217

**Published:** 2011-06-29

**Authors:** LN Markovic-Denic, B Mihajlovic, N Cemerlic-Adjic, K Pavlovic, S Nicin

**Affiliations:** 1 Institute of cardiovascular diseases of Vojvodina, Sremska Kamenica, Belgrade, Serbia; 2 Institute of Epidemiology, School of Medicine, Universite of Belgrade, Belgrade, Serbia

## Introduction / objectives

Needlestick injuries (NSIs) pose a serious risk for health care workers (HCWs). The aim of this study was to examine the impact of an education program on HCWs practices to prevent needlestick and sharp injury.

## Methods

A hospital wide pre- and post interventional study was conducted in a cardiosurgical university hospital. In the first period, the baseline data including NSIs rate, occupation, location, time and activities associated with NSIs were collected using an anonymous questionnaire. During the second period (6 months) an education program was organized through four workshops. HCWs completed the pretest questionnaire at the beginning of the first period (before the first training session) and the post test at the end of last session.

## Results

The questionnaires were answered by 93% of all HCWs in the first period and by 89% at the end of second period. There were 76.1 % female respondents. Their mean age was 38.4 (SD 10.4) with mean total work experience of 15.8 years (SD 10.2). They included 14% physicians, 64% nurses, 3% laboratory technicians, 16% health assistants and 4% of other workers. In the six months before intervention, 13.0% of HCWs had at least one accident. About 40% of HCWs were not vaccinated against hepatitis B. During 6 months of education the NSI rate decreased from 13% to 11.2% (p=0.3). The NSIs because of recapping needles were less often (p=0.04) during the second period. The most frequent location of NSIs was patient’s room (52.5% ca in first and 46% in second period). NSIs significantly decreased during night shift in the second period (p=0.01).

## Conclusion

Training program should be implemented initially to reduce NSIs.

## Disclosure of interest

None declared.

